# Impaired neutralising antibody formation and high transduction efficacy after isolated hepatic perfusion with adenoviral vectors

**DOI:** 10.1038/sj.bjc.6602151

**Published:** 2004-10-12

**Authors:** B van Etten, A M M Eggermont, G Ambagtsheer, S T van Tiel, T L M ten Hagen

**Affiliations:** 1 Erasmus University Medical Centre-Daniel den Hoed Cancer Centre, Department of Surgical Oncology, PO Box 5201, 3008 AE Rotterdam, The Netherlands

**Keywords:** gene therapy, adenovirus, antibodies, liver perfusion

## Abstract

Local adenoviral gene transfer can be performed by means of isolated hepatic perfusion (IHP). This methodology is a very effective and safe way to deliver adenoviral vectors. We studied the immune response after IHP. A decreased neutralising antibody formation was observed, offering possibilities for further research in the field of gene therapy in isolated perfusion settings.

The main reason for failure of adenoviral gene delivery is insufficient transduction efficacy *in vivo*. A method, which improves *in vivo* efficacy, is locoregional administration. Previously, we and others demonstrated successful transduction and subsequent tumour response after isolated limb perfusion (ILP) and isolated hepatic perfusion (IHP) (de Roos *et al*, 1997, 2000; de Wilt *et al*, 2001; van Etten *et al*, 2002).

Adenoviral mediated gene transfer results in transient gene expression. Loss of the therapeutic gene requires readministration to achieve prolonged gene expression, which however is precluded by formation of neutralising antibodies (Yang *et al*, 1994; Kaplan *et al*, 1997). Since IHP is a technique with minimal systemic exposure and washout possibilities after the perfusion, immune response after adenoviral treatment may be impaired.

We performed IHP in rats with a recombinant adenoviral vector and investigated the production of neutralising antibodies and leukocytes compared to systemic intravenous (i.v.) treatment.

## MATERIALS AND METHODS

### Animals

Male inbred immunocompetent WAG/RIJ rats, weighing 250–300 g (Harlan-CPB, The Netherlands) were used. The rats were fed a standard laboratory diet and were housed under standard conditions of light and accommodation. The ethical committee for animal research of the Erasmus University Medical Center approved the protocol. The experimental protocols adhered to the rules outlined in the Dutch Animal Experimentation Act of 1977 and the published UKCCCR Guidelines for the Welfare of Animals in Experimental Neoplasia (Workman *et al*, 1998).

### Recombinant adenovirus construct

The viral constructs were provided by Aventis-Pharma (Vitry-sur-Seine, France) and are described previously by us in detail (van Etten *et al*, 2002). AV1.0CMV is a recombinant replication-deficient adenovirus vector. AV1.0CMV.LacZ expresses the *Escherichia coli* derived *β*-galactosidase protein that can be detected by X-gal histochemistry.

### Routes of administration

#### Isolated hepatic perfusion

We have described the rat isolated liver perfusion model in detail earlier (van IJken *et al*, 2000). Rats were perfused for 10 min with oxygenated and heated (38–39°C) colloid fluid (Haemaccel, Behring Pharma, Amsterdam, Netherlands) with 1.0 × 10^11^ virus particles (vp). This dose was previously determined as the maximum tolerated dose (MTD). Afterwards, a washout procedure was performed to remove all nonbound viruses by perfusing with 10 ml Haemaccel.

#### Intravenous injection

A volume of 200 *μ*l of PBS containing 2.5 × 10^11^ vp (MTD) was slowly injected into the penile vein.

### Blood and tissue sampling

Blood samples were taken via the tail vein at day 0, 3, 6, 9,16, 23, and 30 after treatment. Serum was collected after centrifugation (16 000 × **g**) and stored at −80°C until further analysis. At 24 h after treatment animals were killed. Liver was taken out and snap frozen in liquid nitrogen. Cryosections of tissue samples were stained according to the X-gal staining protocol (van Etten *et al*, 2002).

### Measurement of neutralising antibodies

Adenovirus type 5 specific neutralising antibodies were measured by the virus neutralisation (VN) test as previously described (Schrader and Wigand, 1981; De Jong *et al*, 1999). The presence of cytopathic effects of Hep 2 cells caused by the virus was scored under the microscope. Neutralising antibody titres were expressed as the highest serum dilution showing no cytopathological effects.

### Measurement of leukocyte count, liver and renal functions

Leukocyte numerations were determined with a microcell counter (Sysmex; Kyoto, Japan). Liver functions (alkaline phosphatase, alaline aminotransferase, aspartate aminotransferase, total bilirubin and *γ*-glutamyl transpeptidase) and renal functions (creatinin and urea) were measured by spectophotometric analysis (ELAN-analyzer; Eppendorf-Merck, Hamburg, Germany).

### Statistical analysis

Results were evaluated for statistical significance with the Kruskal–Wallis and Mann–Whitney *U* tests with SPSSv10.0 for Windows 2000. A significance level of *P*<0.05 was used.

## RESULTS

**Figure 1:**
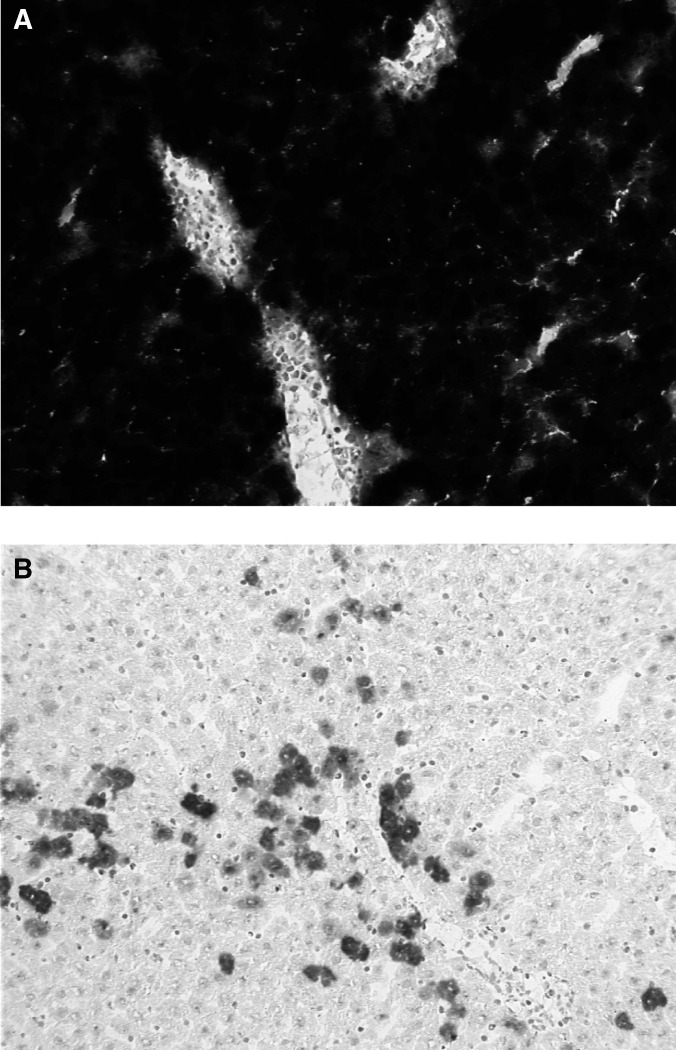
X-gal staining of liver tissue after treatment with AV1.0CMV.LacZ. (**A**) After IHP, estimated transduction of 80–90%. (**B**) After i.v. injection, estimated transduction of about 5%. Representative pictures of liver tissue samples of three animals per treatment.

We observed augmented transfection of cells after isolated perfusion (*n*=3) (transduction efficacy of 80–90%) compared to systemic therapy (*n*=3) (transduction efficacy around 5%) (Figure 1

).

**Figure 2:**
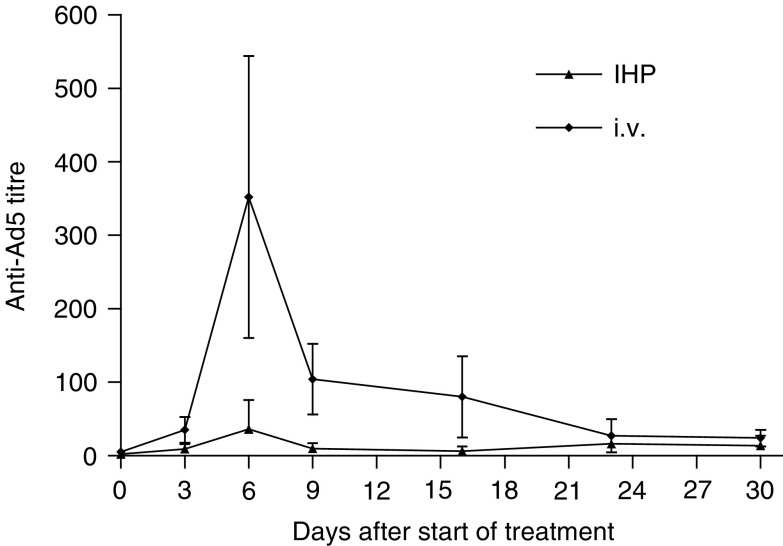
Neutralising antibody titre after i.v. (⧫) or IHP (▴) with AV1.0CMV determined as described in Materials and methods section. Antibody titres after IHP were significantly lower compared to i.v. from day 3 up to day 23 (*P*⩽0.05). On day 6 after treatment a mean peak titre could be measured: 352 after i.v. injection *vs*. 27 after IHP (*P*=0.03). Mean values of five animals±s.d. are shown.

Antibody titres after IHP (*n*=5) were significantly lower compared to i.v. (*n*=5) from day 3 up to day 23 (Figure 2

). On day 6 after treatment a mean peak titre could be measured: 352 after i.v. injection *vs* 27 after IHP (*P*=0.03). During follow-up, performed up to 30 days after treatment the levels decreased and were equalised approximately as from day 23.

Leukocyte count was determined at day 6 after treatment. Numerations of leukocytes (mean values of three animals±s.d.) were significantly higher after i.v. (2.16 × 10^10^±0.19) compared to IHP (1.18 × 10^10^±0.45) or the untreated control group (1.15 × 10^10^±0.16), whereas leukocyte count after IHP was not increased compared to control values (i.v. *vs* IHP: *P*⩽0.05, i.v. *vs* control: *P*⩽0.05). The increased leukocyte count following i.v. administration correlates directly with the rise of titres measured after i.v. treatment.

No severe hepatic or renal toxicity could be detected in the i.v. or IHP group after treatment. Levels of toxicity parameters measured in sera varied in a range of + and −25% of the control values (data not shown). After i.v. injection, no weight loss was observed. Following IHP a maximum transient weight loss of 10% was observed, which could not be related to the viral vector as similar weight loss was observed upon sham IHP.

## DISCUSSION

Isolated hepatic perfusion is a more effective and safe method to deliver adenoviral vectors towards liver or tumour cells compared to i.v. injection (de Roos *et al*, 1997; van Etten *et al*, 2002). A transduction efficacy up to 45% of hepatocytes by means of IHP combined with a chelating agent has been described (de Roos *et al*, 1997). Here we demonstrate that this highly selective delivery method can result in even higher transduction rates and, importantly, is accompanied by an impaired neutralising antibody formation and leukocyte proliferation. Several studies have been conducted to influence immune response upon adenoviral gene therapy, including incorporation of immunosuppressive genes into the vector or manipulation of the immune system during administration (Qin *et al*, 1997; Nakagawa *et al*, 1998; Kuzmin *et al*, 2001). To our knowledge this is the first report demonstrating an impaired immune response using isolated perfusion methodology with a ‘regular’ adenoviral vector in an immunocompetent animal model.

The liver is known for its ability to induce immune tolerance (Calne, 2000). Both in patients and animal models host-to-graft tolerance was observed (Kamada *et al*, 1981; Mazariegos *et al*, 1997). Liver sinusoidal endothelial cells (LSECs), which clear the antigen from the blood can function as an antigen-presenting cell (Limmer *et al*, 2000). Naïve T cells, which are activated by the LSECs, do not differentiate into effector T cells thereby inducing antigen-specific T cell tolerance (Knolle and Limmer, 2001). This mechanism might play a role in the lesser immune response we observed.

It is known that the height of neutralising antibodies titres is correlated with the dose of virus administrated (Yang *et al*, 1996). Since most circulating viruses are washed away at the end the perfusion, only a relative low viral load is left behind in the liver. Worgall *et al* (1997) demonstrated that the acute innate immune mechanism eliminated 90% of the circulating adenoviral vectors within 24 h. So, if hardly any circulating viruses are left after IHP and the majority is cleared rapidly, only an extremely low amount of viruses can induce the humoral immune response, likely resulting in low neutralising antibody production.

Retrograde infusion of adenoviruses in the common bile duct of mice resulted in increased hepatic restricted gene transfer, combined with lower neutralising antibody titres (Peeters *et al*, 1996). It has been reported that the route of administration strongly determines the humoral immunity to the transgene in experiments with adeno-associated virus vectors in mice. Delivery via the hepatic artery resulted in higher transgene expression and an absent immune response to the transgene product (Nathwani *et al*, 2001). These results and our current findings suggest that loco-regional liver directed gene therapy offers advantages at gene transfer efficacy level as well as at immune response level. We recently performed pilot experiments with repeated administration. Primary adenovirus treatment by IHP followed by i.v. challenge 30 days later. These preliminary data showed no advantage with respect to transduction efficacy after the i.v. administration. Since repeated IHP in rats is not possible due to technical reasons we cannot provide data on this issue at this moment. In pigs we previously developed leakage free and potentially repeatable balloon catheter based IHP technique (van IJken *et al*, 1998), offering possibilities for future experiments with repeated IHP.

In conclusion, our findings are a strong argument for further research on delivery of viral vectors in isolated perfusion settings (limb, kidney, lung, liver).
